# Cracking the code on the emergency medicine match: It's about supply and demand, not interviews

**DOI:** 10.1002/aet2.10961

**Published:** 2024-03-21

**Authors:** Alexis E. Pelletier‐Bui, Laura R. Hopson, Jason I. Reminick, Michael C. Bond, Alisa Hayes, Ephy Love

**Affiliations:** ^1^ Department of Emergency Medicine Cooper University Hospital/Cooper Medical School of Rowan University Camden New Jersey USA; ^2^ Department of Emergency Medicine University of Michigan Medical School Ann Arbor Michigan USA; ^3^ Thalamus Santa Clara California USA; ^4^ Department of Emergency Medicine University of Maryland School of Medicine Baltimore Maryland USA; ^5^ Department of Emergency Medicine, Medical College of Wisconsin Milwaukee Wisconsin USA

## THE 2022 AND 2023 EMERGENCY MEDICINE MATCHES

Emergency medicine (EM), a historically highly competitive specialty, experienced an abrupt change in the National Resident Matching Program (NRMP)'s Main Residency Match (hereafter referred to as “The Match”) results in 2022 and 2023. Unfilled residency positions increased from an average of 0.48% (2012–2021) to 7.4% (2022) and 18.4% (2023), leaving 46% of EM residency programs facing vacancies in 2023.^1^

This drastic shift produced keen scrutiny to the cause. Potential factors fall into three areas: excess supply of positions, lack of student demand for EM and problems embedded in the recruitment process.

Key insights into EM's current challenges can be gleaned from publicly available data from the Electronic Residency Application Service (ERAS), the NRMP, and the Accreditation Council for Graduate Medical Education (ACGME) as well as data from Thalamus (a graduate medical education interview management platform representing approximately 25% of EM programs; “Thalamus”) and additional NRMP data as a result of a data sharing agreement with Thalamus (“NRMP/Thalamus”).

## PIPELINE PROBLEMS: EXCESS SUPPLY AND DECREASED DEMAND

EM programs in The Match increased from 170 to 287 (69%) from 2014 to 2023,^1^ which includes 50 American Osteopathic Association programs that transitioned to the ACGME.^2^ EM positions increased from 1786 to 3010 (69%) over the same period through both contribution from new programs and expansion of existing programs.^1^, ^2^

**FIGURE 1:**
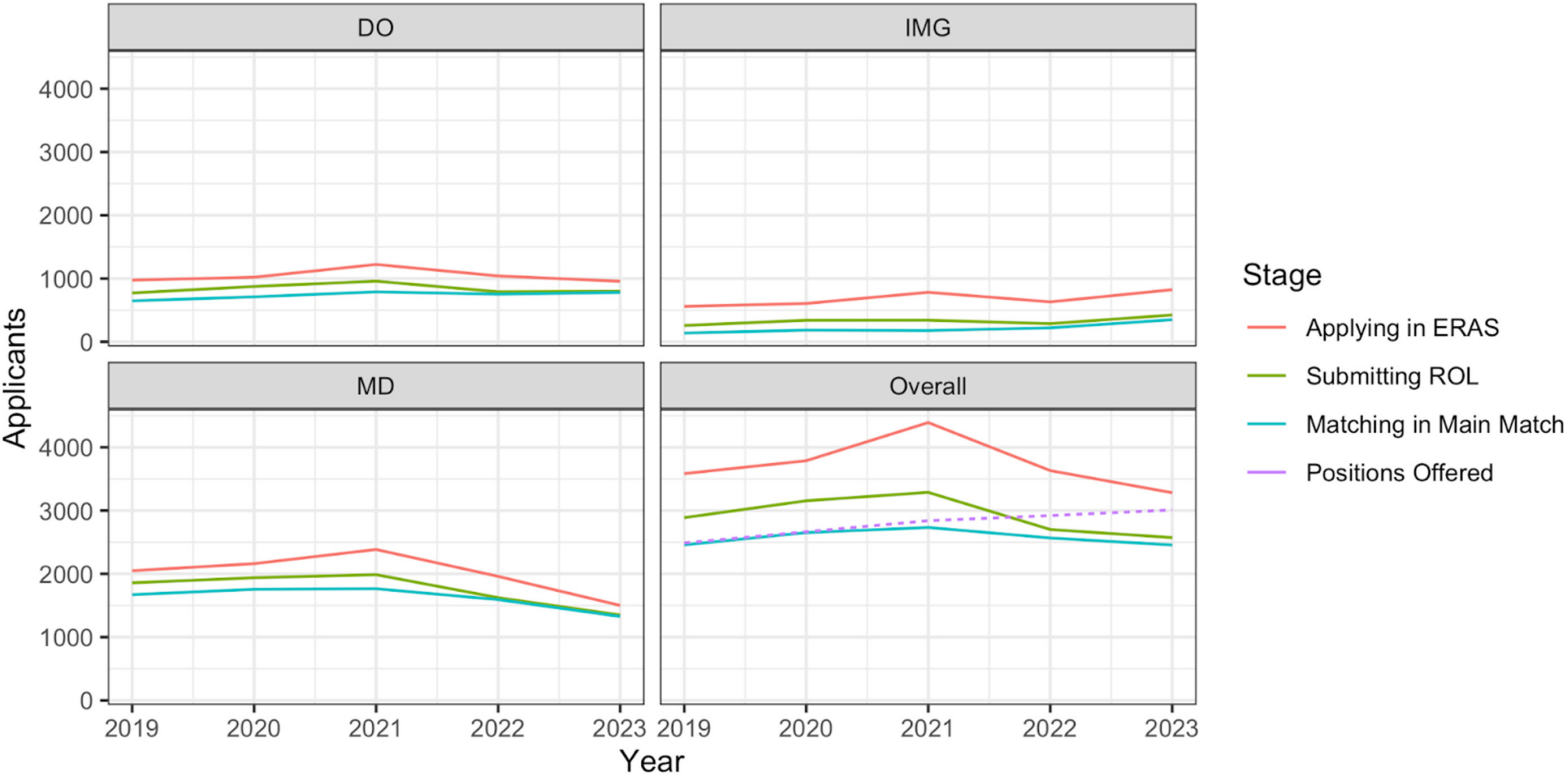
Number of EM applicants from 2019 to 2023 by applicant type and residency application process stage. DO, osteopathic; ERAS, Electronic Residency Application Service; IMG, international medical graduate; MD, allopathic; ROL, rank order list.

After a steady increase in applicants from 2019 to 2021, allopathic and osteopathic applicants decreased substantially in 2022 and 2023, with the steepest decline in allopathic applicants (Figure 1). Total applications from all applicant types declined by approximately 17% year‐over‐year for the last two Match cycles (email communication from ERAS Strategy & Engagement Director, Michele Oesterheld, May 2023). In both 2022 and 2023, the number of applicants preferring EM who submitted a rank order list (ROL) in EM was lower than the number of positions available (NRMP/Thalamus; Figure 1).

In sum, almost every 2023 EM applicant in ERAS would have needed to match in EM to fill the available positions, which was unrealistic. For the past 2 years, approximately 30% of applicants to EM also applied to at least one other specialty (email from ERAS Pilot Administration Director, Jayme Bograd, October 2022), with some preferring that other specialty (NRMP/Thalamus). Other applicants may have had academic challenges, visa, licensing, or credentialing issues that hindered their consideration at many institutions. These data clearly illustrate that a supply‐and‐demand mismatch exists between EM applicants and currently available positions.

## INTERVIEW BEHAVIORS

Some propose that potentially modifiable program and applicant interview behaviors contribute to Match results.^3^, ^4^ These are likely limited in their impact.

### Interview hoarding

Applicant interview hoarding, meaning a small cohort of applicants taking a large proportion of available interviews, could prevent programs from having equal opportunities to interview and rank applicants.^5^ This concern has led to calls for interventions including application and interview caps, universal interview days, program signaling, and an early Match.^3^

However, current data suggest that interview hoarding is not a driver of EM unmatched programs. The average ROL length for applicants preferring EM only minimally increased from 17.2 (2019–2020) to 18.3 (2021–2023) with virtual interviews. Mean applicant interview overlap (MAIO) was 9.82% in 2021, 10.53% in 2022, and 12.04% in 2023 (Thalamus). Therefore, for any two randomly selected programs, an average of 12% of their interviewees would be common. These values are consistent with specialties of comparable size, and some overlap is expected due to programs with similar geographic and program characteristics. The increase in MAIO from 2021 to 2023 is also expected given a smaller applicant pool each year, as fewer applicants mean a greater likelihood of overlap as interview positions remain relatively fixed. Overall, consistent applicant ROL length combined with a minimal increase in the MAIO suggest that EM likely has an appropriate level of overlap, arguing strongly against a small number of applicants compromising the global Match results.

### Interviewing the “wrong” candidates

Another hypothesis is that unfilled programs interviewed the wrong candidates who were unlikely to be high‐yield matches due to credentialing or geographic mismatches.^4^ EM leaders encouraged programs to diversify their pool of candidates through further consideration of osteopathic or international medical graduates (IMGs).^4^ EM programs responded based on recent increased application to interview conversion rates for all applicant types (NRMP/Thalamus). Application to interview conversion rates from 2021 to 2022 to 2023 were as follows: 37%–37%–47% (allopathic), 25%–26%–35% (osteopathic), and 7%–10%–12% (IMG). IMG application to interview conversion demonstrated the largest relative increase during these Match cycles. However, based on the lower number of IMG applicants to EM, further expansion of an IMG pool could be required to decrease unfilled positions.

### Interviewing too few applicants

Some hypothesize that unfilled programs interviewed too few applicants.^4^, ^6^ Programs interviewed 3.3% more candidates in 2023 versus 2022 (Thalamus). Due to a diminishing number of applicants, “interviewing more” still does not change the specialty outcome. In other words, each EM program could have interviewed every single applicant who preferred EM in 2023, and still nearly 400 positions would have gone unfilled (NRMP/Thalamus).

## SOLUTIONS

We need to understand *why* the pipeline to EM is decreasing. One major concern is the projected surplus of 7845 EM physicians by 2030 limiting future job prospects.^7^ While there will still be EM job shortages in many areas,^8^ geography drives most individuals' decisions when choosing where to train and practice.^1^ Whether or not the assumptions around the initial workforce study are sustained,^8^ the impact of the initial report will likely not rapidly abate.

Anesthesiology previously experienced similar workforce challenges. From the late 1980s to early 1990s, anesthesiology residency positions exploded. A 1994 workforce assessment predicted a future oversupply. Extensive publicity drove a precipitous decline of U.S. applicants.^9^ By 2000, IMGs comprised more than half of the graduating anesthesiologists in the United States.^9^ Total applicants to anesthesiology did not begin to recover until 2001 data forecasted a significant workforce shortage. Reassessment of the anesthesiology workforce in 2011 showed that entry rates into anesthesiology still remained below 1993 levels and projected continued workforce shortages due to further contraction of training positions.^9^ Extrapolating from anesthesiology, it is unlikely that student interest in EM will increase until they are confident of future job availability.

### Contraction of programs

EM's solution may be the contraction of residency programs. Does the United States need 8% of graduating medical students going into EM?^1^ As national physician shortages across most other disciplines are projected,^10^ EM needs to grapple with the difficult question of whether these unfilled positions in EM should be allocated to other specialties to better represent societal needs.

While we may hope that market forces will bring things into balance, all but 44/545 unfilled 2023 EM positions were eventually filled through the Supplemental Offer and Acceptance Program (SOAP) process.^1^ Therefore, it is unlikely that programs unfilled prior to the SOAP will contract or close. The contraction of positions may require collective action from all EM programs. Existing programs should consider contraction or at least not expand. New programs should not open unless in geographic areas with a dearth of EM physicians. Programs that recurrently go unfilled should reconsider their complement and training priorities. While the contraction of programs may be a potential solution, EM must also be careful not to overcorrect and be faced with future shortages.

### Policy changes

In the short term, institutional policies and cultures that typically curtail residency programs from considering osteopathic or IMG candidates will need to change in order to fill EM's positions. Legislative changes allowing for national oversight of EM positions by an existing or new organization may also be required given the current lack of authority for ACGME or specialty organizations to regulate EM positions.

### Recruitment of applicants to EM

In addition to future employment concerns, high levels of clinician burnout and work environment concerns are prominent.^6^ EM needs to address the underlying features that may be driving students away. The specialty must share with applicants and the public the numerous benefits and societal needs of a highly trained and specialized EM workforce.

## CONCLUSIONS

The excess supply of emergency medicine positions and lack of student demand are the primary drivers for the 2022 and 2023 emergency medicine Match experiences. Modifying interview behaviors will not resolve the situation. Improvement in future Match cycles will require a change in student interest. Emergency medicine must address its detractions including workforce projections and the work environment. We must educate applicants on the numerous benefits of selecting a career in emergency medicine. We will need to support our training programs through difficult decisions regarding program size and tackle institutional and national policy changes.

## AUTHOR CONTRIBUTIONS

Alexis E. Pelletier‐Bui: Study concept and design, drafting of the manuscript, critical revision of the manuscript for important intellectual content, analysis and interpretation of the data. Laura R. Hopson: Study concept and design, drafting of the manuscript, critical revision of the manuscript for important intellectual content, analysis and interpretation of the data. Michael C. Bond: Study concept and design, drafting of the manuscript, critical revision of the manuscript for important intellectual content, analysis and interpretation of the data. Alisa Hayes: Study concept and design, drafting of the manuscript, critical revision of the manuscript for important intellectual content, analysis and interpretation of the data. Jason I. Reminick: Study concept and design, drafting of the manuscript, critical revision of the manuscript for important intellectual content, acquisition of the data, analysis and interpretation of the data. Ephy Love: Study concept and design, drafting of the manuscript, critical revision of the manuscript for important intellectual content, acquisition of the data, analysis and interpretation of the data, statistical expertise.

## CONFLICT OF INTEREST STATEMENT

LRH reports the following conflicts of interest: Research support from Toyota Motor Co. for unrelated work (PI‐Brent, University of Michigan, ended August 2022), Member of CORD BoD and this work does not reflect organizational opinions. JIR and ERL report the following conflicts of interest: JIR and ERL are both shareholders and employees in Thalamus. The other authors declare no conflicts of interest.
